# Percutaneous Closure of Left Atrial Appendage affects Mid-Term Release of MR-proANP

**DOI:** 10.1038/s41598-017-08999-4

**Published:** 2017-08-22

**Authors:** Michael Behnes, Benjamin Sartorius, Annika Wenke, Siegfried Lang, Ursula Hoffmann, Christian Fastner, Martin Borggrefe, Thomas Roth, Jakob Triebel, Thomas Bertsch, Ibrahim Akin

**Affiliations:** 1 First Department of Medicine, University Medical Center Mannheim (UMM), Faculty of Medicine, Mannheim, University of Heidelberg, Mannheim, Germany; 2 Institute of Clinical Chemistry, Laboratory Medicine and Transfusion Medicine, General Hospital Nuremberg, Paracelsus Medical University, Nuremberg, Germany; 3 Central Laboratory, University Hospital Erlangen, Erlangen, Germany

## Abstract

The left atrial appendage (LAA) represents both a predisposing source of thrombus formation and of neuro-humoral haemostasis. This study aims to evaluate changes of biomarker expression before and after successful percutaneous closure of the LAA. Patients with atrial fibrillation and contraindication for oral anticoagulant therapy were enrolled. Blood samples were taken within 24 hours before (T1) and at least 6 months (mid-term) (T2) after successful implantation of LAA occlusion devices. Blood levels of high sensitivity troponin I and T (hsTnI, hsTnT), aminoterminal pro-brain natriuretic peptide (NT-proBNP) and mid-regional pro-atrial natriuretic peptide (MR-proANP) were evaluated at both time points. A total of 42 patients with successful percutaneous LAA closure were included. Median mid-term follow-up was of 183 days. HsTnT, hsTnI and NT-proBNP did not show any significant differences over time. Serum levels of MR-proANP increased significantly between immediate pre-intervention (T1: median = 245.7 pmol/l, IQR 155.8–361.3 pmol/l) and at mid-term follow-up (T2: median = 254 pmol/l, IQR 183.4–396.4 pmol/l) (p = 0.037). These results indicate, that percutaneous LAA closure affects neuro-humoral haemostasis by increasing MR-proANP serum levels at mid-term follow-up.

## Introduction

Usually biomarker research in cardiovascular diseases has focused on coronary heart disease and heart failure. Examples for biomarkers, that have found the path from the work bench to clinical routine are natriuretic peptides (BNP, NT-proBNP, ANP, MR-proANP) as well as newly developed high sensitivity troponins T and I (hsTnT, hsTnI)^1–3^.

From the perspective of a cardiologist the left atrial appendage (LAA) represents a central part of the heart. Multi-pathological effects make the LAA a pre-disposed source for thrombus formation – besides the left atrium or ventricle – in patients suffering from atrial fibrillation, which is responsible for the development of central or peripheral thromboembolisms^4^. However, the LAA additionally represents a source of neuro-humoral haemostasis, although to what extend is so far mostly unknown and under ongoing debate. Modern biomarker research aims to illustrate the neuro-humoral status of the heart and ideally might be able to reflect the multi-pathological processes cardiac diseases force onto the individual^5^. Cardiovascular biomarkers play an important role to access the neuro-humoral state of the heart, which already has emerged toward a decisive factor in the therapeutic decision in patients with heart failure or coronary heart disease^2, 3^.

Atrial fibrillation comes alongside with the need for oral anticoagulation to prevent LAA thrombus formation that may cause life threatening ischemic stroke^6^. Contraindication for oral anticoagulants such as recurring bleedings bring up a need for an alternative therapy. In this case, percutaneous LAA closure is the recommended option^6–9^. But it has never been evaluated how this iatrogenic percutaneous intervention does influence the neurohormonal state of the heart after successful LAA closure.

It has been shown, that the LAA has its place in the production of natriuretic peptides (ANP)^10–12^, whereas a relation to the secretion of cardiac ischemia markers has so far not been analysed. The “Left Atrial Appendage Occlusion and Biomarker Evaluation” (LABEL) study aims to label the neuro-humoral changes after percutaneous LAA closure by evaluating several biomarkers to gain more pathophysiological insights. This special report evaluates some of the most referenced cardiac biomarkers - NT-proBNP, MR-proANP, hsTnT, hsTnI – before and after successful percutaneous closure of the LAA.

## Methods

The “Left Atrial Appendage Occlusion and Biomarker Evaluation” (LABEL) study (ClinicalTrials.gov Identifier: NCT02985463) is a single-centre, prospective, observational non-randomized study including patients being eligible for percutaneous LAA closure according to European guidelines^6^. All patients presented with non-valvular atrial fibrillation, a CHA2DS2-Vasc score ≥2, a HASBled score ≥3 and a contraindication for the therapy with oral anticoagulants, i.e. major or recurring bleedings.

Exclusion criteria included age <18 years, congestive heart failure classified as NYHA III and IV, catheter ablation of atrial fibrollation within 30 days prior to planned intervention, myocardial infarction within the last 3 months, atrial septum defect or implanted ASD occluder, mechanical heart valves, conditions with increased thrombus formation risk, such as severely reduced LVF or presence of LAA or LV thrombus, prior deep vein thrombosis or pulmonary embolism, status after heart transplant, symptomatic carotid artery stenosis, transient ischemic attack or stroke within 3 months, existing or planned pregnancy, acute renal failure, exacerbated COPD, acute infection or any thrombus at the day of planned implantation. Patients were excluded when revealing acute cardiovascular symptoms, such as angina pectoris, dyspnoea or peripheral oedema.

The study was carried out according to the declaration of Helsinki and was approved by the medical ethics committee II of the Faculty of Medicine Mannheim, University of Heidelberg, Germany. Written informed consent was obtained from all patients or their legal representors.

Percutaneous closure of the LAA was performed using either the Watchman (Boston Scientific, Marlborough, MA, USA) or Amplatzer Amulet (St. Jude Medical, St. Paul, MN, USA) device. Blood samples were taken by venous puncture within 24 hours prior to cardiac intervention (T1). Secondary blood samples were taken at least 6 months later (i.e. mid-term) (T2). At the time of blood sampling all patients had to be in clinical stable conditions, e.g. not suffering from symptoms like angina pectoris, dyspnoea, peripheral oedema, acute renal failure, exacerbated COPD or acute infection. Successful closure of LAA was confirmed by transoesophageal echocardiography (TEE) during index procedure, as well as at mid-term follow-up by TEE and cardiac computed tomography angiography (CCTA)^13^.

All relevant clinical data regarding prior medical history, medication (such as beta blocker, ACE inhibitor, aldosterone antagonists, diuretics), actual symptoms and laboratory data were documented in detail both at baseline as well as follow-up.

Venous blood samples were taken from each patient and collected into serum monovettes® and EDTA monovettes® and centrifuged at 2500 x g for 10 minutes at 20 °C. The aliquoted samples were cooled down with liquid nitrogen before being stored at −80 °C until analysis. The whole processing took part within two hours after blood extraction. After thawing the samples were mixed gently by inverting and centrifuged at 2500 x g for 10 minutes at 20 °C for Troponin T and NT-proBNP analysis.

Troponin T was measured with the Troponin T hs STAT assay on a cobas e 602 analyser (Roche Diagnostics, Mannheim, Germany). The limit of blank (LoB) for this assay was 0.003 ng/mL and the Limit of Detection (LoD) 0.005 ng/mL as described in the instructions for use. For TnI measurement the samples were gently mixed by inverting after thawing and centrifuged for 30 minutes at 3000 x g at 4 °C.

Troponin I was measured with the STAT High Sensitive Troponin-I assay on an Architect i1000 analyser (Abbott, Wiesbaden, Germany) with a LoB of 0,7–1,3 pg/mL and a LoD of 1,1–1,9 pg/ml. NT-proBNP was measured with the proBNP II STAT assay on a cobas e 602 analyser (Roche Diagnostics, Mannheim, Germany). The LoD for this assay was 5 pg/mL.

MR-proANP was measured in EDTA-plasma on a Kryptor compact plus analyzer (Thermo Scientific B.R.A.H.M.S., Henningsdorf, Germany). Prior analysis the plasma was tawned, gently mixed and centrifuged at 2700 x g for 15 minutes at 20 °C.

Statistical analysis comprised the following calculations: For normally distributed data the student t test was applied and changes of biomarkers expression over time, i.e. in between T1 and T2, were analysed by paired t-tests. Qualitative parameters were analysed using the Chi^2^ or Fisher’s exact test. In all cases a p value of <0.05 was utilized for statistical significance. Statistical analyses were performed with SPSS and GraphPad Prism.

## Results

A total of 42 patients with successful percutaneous closure of the LAA and complete mid-term follow-up were included from April 2014 until November 2015. Baseline characteristics and their procedural data are shown in Table 1. Slightly more than half of patients (55%) received a Watchman and 45% an Amplatzer cardiac plug or amulet device. All patients revealed successful percutaneous LAA closure being assessed during intervention and at mid-term follow-up with complete lobe coverage and no peri-device leaks of more than 5 mm.

**Table 1:** Baseline characteristics of 42 patients with successful left atrial appendage occlusion and biomarker evaluation.

Characteristic	Value
**Demographics**	
Sex, male n (%)	29 (69)
Age, y (IQR)	76.7 (74–82)
Height, cm (IQR)	170.8 (166–176)
Weight, kg (IQR)	82.6 (71.4–92)
BMI (IQR)	28.2 (24.7–32.9)
**Cardiovascular risk factors, n (%)**	
Hypertension	40 (95)
Diabetes mellitus	15 (36)
Hypercholesterinemia	20 (48)
**Medical history, n (%)**	
Atrial fibrillation, n (%)	
paroxysmal	22 (52)
persistent	6 (14)
permanent	14 (33)
Prior PVI, n (%)	4 (10)
TIA, n (%)	2 (5)
Stroke, n (%)	6 (14)
Coronary artery disease, n (%)	23 (55)
Prior myocardial infarction, n (%)	8 (19)
Heart failure	8 (19)
Peripheral vascular disease, n (%)	4 (10)
Chronic kidney disease, n (%)	16 (38.1)
Creatinine, mg/dl (IQR)	1.06 (0.94–1.3)
MDRD-GFR, ml/min (IQR)	63.9 (50.6–79.7)
Chronic liver disease, n (%)	3 (7)
Prior bleeding, n (%)	32 (76)
CHA_2_DS_2_-VASc score (IQR)	4 (3–5)
HAS-BLED score (IQR)	3.6 (3–4)
**Events at mid-term follow-up, n (%)**	
Acute myocardial infarction	1 (2)
Stroke	0 (0)
Pulmonary embolism	1 (2)
Bleeding	7 (17)
Rehospitalization	23 (55)
Cardio-pulmonary diseases	11 (26)
Bleedings	7 (17)
Musculosceletal syndrome	3 (7)
Others	3 (7)

Values are given as median (25^th^ and 75^th^ percentiles) or total numbers (percentage). AF = atrial fibrillation, PVI = pulmonary vein isolation, TIA = transient ischemic attack, AMI = acute myocardial infarction.

Blood samples for biomarker measurements were obtained from all patients at T1 and T2 within a median follow-up of 183 days (interquartile range (IQR), 174–207 days) after successful percutaneous closure of the LAA. 55% patients were re-hospitalized during follow-up. Events between both time points are listed in Table 1. Major adverse cardiovascular events were documented in 2 patients: one patient suffered ST segment elevation myocardial infarction (STEMI) and underwent percutaneous coronary intervention (PCI). Another developed pulmonary embolism one month after LAA closure and oral anticoagulation with Rivaroxaban was re-initiated. Both events occurred at least 3 months before second blood samples were taken. Patients´ medication influencing volume status, electrolytes or sympathetic activation, such as diuretics, beta-blockers or ACE/aldosterone antagonists, did not change in type or dosage in between baseline and follow-up.

**Figure 1:**
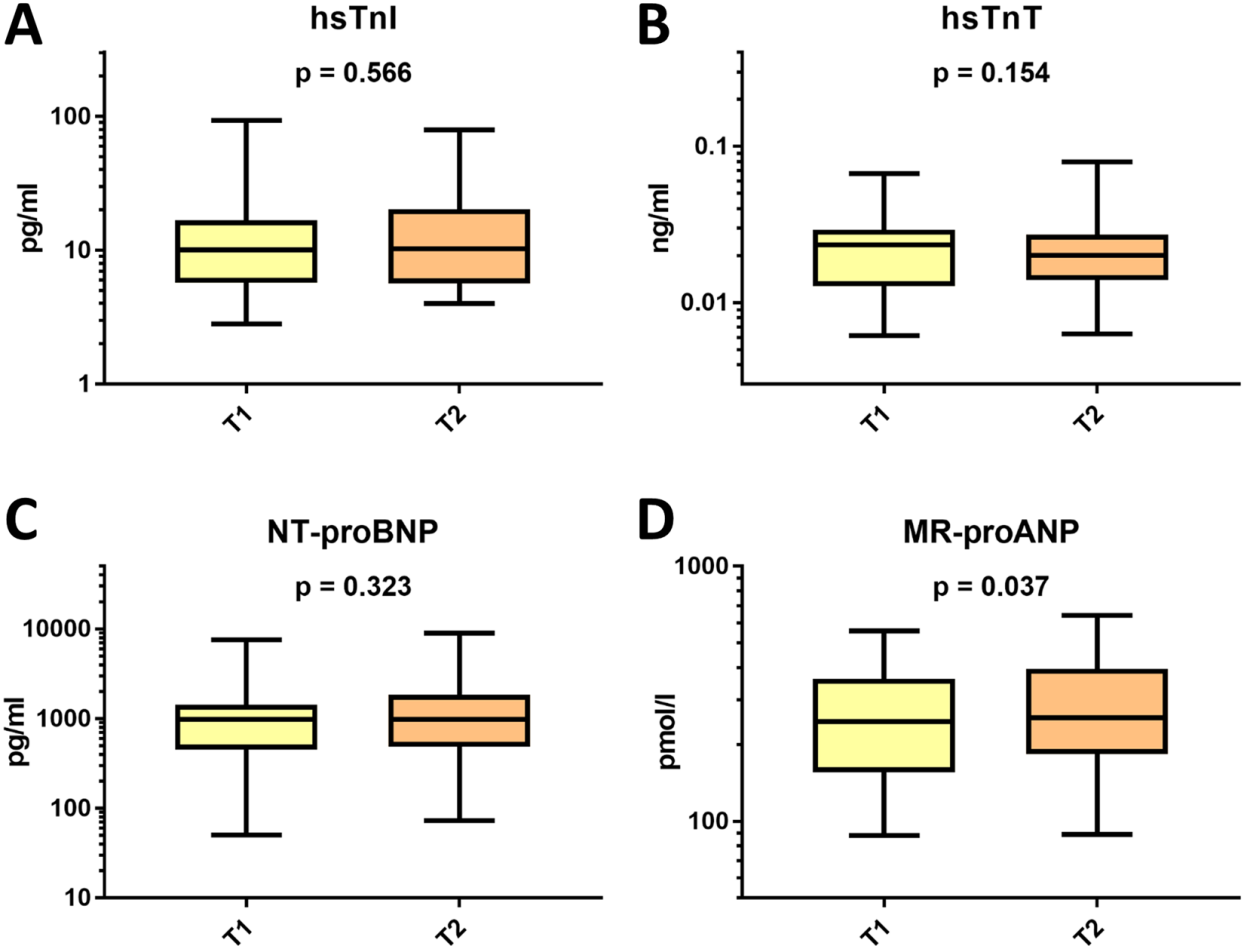
Box plots illustrating changes of the four cardiac reference biomarkers 24 hours prior to (T1) and at mid-term follow-up after (T2) successful percutaneous closure of the LAA (T2): panel A, hsTnT; panel B, hsTnI; panel C, NT-proBNP; panel D, MR-proANP. *P* *<* *0.05 indicates statistical significance. Bars indicate medians, boxes indicate 25*–*75. percentiles, and whiskers indicate 5* ^*th*^–*95* ^*th*^ *percentiles*.

As demonstrated in Fig. 1, none of the currently known reference biomarkers, such as NT-proBNP and both high sensitivity troponins (I and T), changed over time before and after successful percutaneous closure of the LAA at mid-term follow up (Fig. 1A–C, Table 2) In contrast, MR-proANP levels increased significantly (T1: median 245.7 pmol/l, IQR 155.8–361.3 pmol/l; T2: 254 pmol/l, IQR 183.4–396.4 pmol/l; p = 0.037; Fig. 1D, Table 2).

**Table 2:** Biomarker values of 42 patients at T1 and T2 after successful left atrial appendage occlusion.

Biomarker, median (IQR)	T1	T2	p value
hsTnI, pg/ml	10.1 (5.7–16.8)	10.3 (5.7–20.3)	0.566
hsTnT, ng/ml	0.024 (0.013–0.029)	0.02 (0.014–0.027)	0.154
NT-proBNP, pg/ml	975.3 (455.2–1429)	981.4 (488–1852)	0.323
MR-proANP, pmol/l	245.7 (155.8–361.3)	254 (183.4–396.4)	**0.037**

Values are given as median (25^th^ and 75^th^ percentiles). T1 = within 24 h prior to LAA closure, T2 = mid-term follow-up after LAA closure.

Glomerular filtration rates decreased numerically from T1 to T2 (T1: median 65.5 ml/min; IQR 50.6–79.7 ml/min; T2: median 59.2 ml/min; IQR 40.7–79.6 ml/min; p = 0.056). Also, MR-proANP correlated significantly with GFR both at T1 and T2 (p = 0.0001). However after adjustment, the change of GFR over time (ΔGFR) did not significantly influence the increase of MR-proANP levels over time (ΔMR-proANP) (p value for interaction = 0.130).

Patients treated with Watchman occlusion devices showed an average MR-proANP increase of 37.5 pmol/l, compared to 7.5 pmol/l with ACP devices (p = 0.091). Transesophageal echocardiographic (TEE) data are given in Table 3. No changes of left ventricular function, wall thicknesses or left atrial size were found in between baseline and follow-up (data not shown). At follow-up (T1), the TEE-based assessment revealed minor peri-device leaks in 15%, minor atrial septal defects (ASD) / persistent foramen ovale (PVO) in 18% after trans-septal puncture at baseline, and slight pericardial effusion in only 1 patient. No significant differences of any biomarker were seen in any of these subgroups over time (p > 0.05).

**Table 3:** Trans-esophageal echocardiography data at baseline (T1) and follow-up (T2).

Characteristics	Value
**Baseline, median (interquartile range (IQR))**	
Septum, mm	12 (11–14)
LVEDD, mm	48 (43.5–52.3)
Left ventricular function	
Normal (>55%)	34 (81)
Slightly reduced (≥45–55%)	4 (9.5)
Moderately reduced (≥35–45%)	4 (9.5)
Severely reduced (<35%)	0
Left atrial diameter (LAD), mm	48 (43.8–54.5)
Left atrial volume (LAV), mm³	83.5 (66.3–103.3)
LA diameter, mm	22 (19–25)
LAA orifice, mm	
45°	18 (17–21)
90°	19 (17–21)
135°	20 (18–22)
LAA depth, mm	29 (25–35)
LAA landing zone, mm	19 (16–22.5)
**Follow-Up**	
Device compression, %	0.8 (0.8–0.9)
ASD/PFO, n (%)	7 (17.5)
Peri-device leaks, n (%)	6 (15)
Pericardial effusion, n (%)	1 (2.5)
Device thrombus, n (%)	0

LA = left atrium, LAA = left atrial appendage, LVEDD = left ventricuar end diastolic diameter, LVF = left ventricular function, ASD = atrial septal defect, PFO = patent foramen ovale.

## Discussion

The LABEL study demonstrates a significant increase of blood levels of MR-proANP at mid-term follow-up after successful percutaneous LAA occlusion. In contrast, currently known blood-derived cardiac reference biomarkers, such as NT-proBNP or high sensitivity troponin I and T, did not reveal any changes within mid-term follow-up.

The presented results might generate new hypotheses regarding the influence of interventional LAA closure on the neuro-humoral pathophysiology of the heart for further investigations. The LAA represents the main source of ANP production^4^, however whether mid-regional fragment might be influenced after interventional LAA closure at mid-term follow-up has never been investigated before^12^. This might also explain the unchanged expressions of NT-proBNP and hs troponins, since all patients were in a stable cardiac condition. The prevalence of compensated chronic heart failure was 19% and 2 acute coronary syndrome developed within the follow-up period.

The significant increase of MR-proANP might be explained by permanent distension of the LAA occlusion device resulting in mechanical stress at the LAA landing zone. The device compression guarantees permanent and stable positioning for the device life-long with a recommended compression rate of 10–15%^14^. The permanent iatrogenic stretch in turn mimicking volume or pressure overload might be the stimulating factor for the release of MR-proANP at mid-term follow-up. It has been shown recently, that several situations do occur within the LAA itself after percutaneous closure^13^. Within mid-term follow-up LAA occlusion devices are not re-endothelialized completely, which might be detected by indirect signs of permanent contrast enhancement within the LAA being assessed by CCTA^13^. Additionally, minor to moderate peri-device leaks of less than 3 mm are accepted and a residual lobe of less than 1 cm have been described in the literature^13^. Furthermore, ongoing contraction of the LAA itself is still observed at mid-term follow-up despite successful percutaneous closure. These aspects in combination with increasing levels of MR-proANP at mid-term follow-up may reflect ongoing pathophysiologic alterations of the LAA within the cardiac cycle. The LAA occlusion device does not lead to an obliteration or loss of function of the LAA, whereas it needs to achieve comparable protection from thrombus embolization from the LAA to prevent ischemic stroke in patients with atrial fibrillation^6^.

The findings of the present study are observational and reveal hypothesis generating character. To what extend the increase of MR-proANP levels due to percutaneous LAA closure might affect the clinical disease course of patients at long-term follow-up needs to be speculated. In the present study cohort worsening of heart failure symptoms or cardiopulmonary exercise capacity was not evident, and therefore a significant impact at mid-term follow-up may not be clinically ouvert. However, an increase of MR-ANP was shown to reveal both positive and negative effects. ANP increases diuresis and natriuresis by inhibiting the renin-angiotensin system^15^, which was also considered as a pharmacological approach^16^. On the other hand, increases of MR-proANP were associated with an impaired long-term mortality in patients suffering from chronic heart failure^17^. Whether or not LAAC-associated MR-proANP increase might reveal either beneficial or adverse prognostic effects beyond preventing systemic thromboembolism needs to be evaluated further within experimental and clinical studies.

### Limitations

The present results are based on a small sample size and need to be re-evaluated in larger study populations and several subgroups with an even longer follow-up period to determine the long-term effects on neuro-humoral haemostasis after LAAC. Further investigation need to combine imaging results with biomarker expression to explain alterations of biomarkers with morphological signs of incomplete LAA closure or compressions of neighbouring structures. To what extend other biomarkers might be influenced by percutaneous LAA closure will be evaluated further on within the present LABEL and comparable studies.

### Data availability

The main data supporting the present findings is contained within the manuscript or referenced. The related dataset can be obtained directly from the author upon reasonable request.
